# Association between Fruit and Vegetable Intake and Physical Activity among Breast Cancer Survivors: A Longitudinal Study

**DOI:** 10.3390/curroncol28060422

**Published:** 2021-11-30

**Authors:** Steve Amireault, Jennifer Brunet, Jordan D. Kurth, Angela J. Fong, Catherine M. Sabiston

**Affiliations:** 1 Department of Health and Kinesiology, Purdue University, West Lafayette, IN 47907, USA; kurth1@purdue.edu; 2 School of Human Kinetics, University of Ottawa, Ottawa, ON K1N 6N5, Canada; jennifer.brunet@uottawa.ca; 3 Section of Behavioral Sciences, Rutgers Cancer Institute of New Jersey, New Brunswick, NJ 08903, USA; angela.fong@rutgers.edu; 4 Faculty of Kinesiology and Physical Education, University of Toronto, Toronto, ON M5S 2W6, Canada; catherine.sabiston@utoronto.ca

**Keywords:** breast neoplasms, diet, exercise, longitudinal studies, survivors

## Abstract

This study examines the association between rates of change in daily fruit and vegetable intake and in weekly levels of moderate-to-vigorous intensity physical activity (MVPA) over a 15-month period in women following primary treatment completion for breast cancer. Breast cancer survivors (*N* = 199) self-reported fruit and vegetable intake and wore an accelerometer for 7 consecutive days to measure levels of MVPA on five occasions every 3 months. Multivariate latent growth modeling revealed that the rate of change in fruit and vegetable intake was not associated with the rate of change in levels of MVPA. Baseline (Mean = 3.46 months post-treatment) levels of MVPA were not associated with the rate of change of daily fruit and vegetable intake; likewise, baseline fruit and vegetable intake was not associated with the rate of change in levels of MVPA. Behavioral interventions promoting fruit and vegetable intake should not be assumed to yield concomitant effects in promoting MVPA or vice versa.

## 1. Introduction

Unhealthy dietary behaviors and physical inactivity are common in adults during and after treatment for cancer [1,2,3,4,5]. Evidence suggests that people who typically eat less fruits and vegetables also typically engage in less moderate-to-vigorous intensity physical activity (MVPA) [1,2,3,4,5]. This is a clinically important issue because the combined effect of both behaviors on quality of life [1,2,6] and cancer-specific and all-cause mortality [5,7,8] is greater than the sum of their separate effects. Additionally, the co-occurrence of these behaviors can lead to excessive weight gain and obesity, which can further increase the risk of morbidity, cancer recurrence, cancer treatment-related symptoms (e.g., functional limitation, lymphedema, cancer-associated thrombosis) [9,10,11], and cardiovascular disease and cardiovascular disease-related death [12,13]. Accordingly, there is growing interest in behavior change interventions that target both dietary and physical activity behaviors to reduce these risks and improve overall health and well-being among cancer survivors [14,15].

Before designing interventions that target both dietary and physical activity behaviors, however, a better understanding of the covariance of these two behaviors over time is needed. Such knowledge would provide insights as to whether behavior change interventions targeting one behavior could potentially lead to improvement in the other and whether behaviors should be targeted simultaneous or sequentially [16,17]. There are two main relevant theoretical perspectives that would support the covariation of health-promoting behaviors over time [18,19]. First, the multiple behavioral (or carry-over) hypothesis suggests that past successes engaging in one health-promoting behavior may serve as a gateway to changing another health-promoting behavior [20,21,22]. Specifically, the compensatory carry-over action model [18] posits that people can carry over resources, experiences and use of self-regulation strategies from one health-promoting behavior to another. Second, the higher-level goals (or global health) hypothesis suggests that higher-level goals, especially those that are emotionally relevant and require the adoption of multiple behaviors to increase the chances of success (e.g., to change body weight, reduce risk of cancer recurrence and cardiovascular disease), can motivate the adoption of two or more specific behaviors. Although application of these two theoretical perspectives is limited [20,21,22], they nonetheless both suggest that cancer survivors’ early fruit and vegetable intake and early engagement in MVPA may go beyond helping to increase or maintain these respective behaviors, but affect their participation in other health behaviors.

Cross-sectional studies investigating changes in dietary intake or physical activity indicate that most cancer survivors report increases in consumption of fruit and vegetable (50–85%), whereas most report maintaining (~40–50%) or decreasing their levels of physical activity (~25–30%) since their cancer diagnosis [23,24,25,26]. Likewise, longitudinal studies report that breast cancer survivors tend to engage in less physical activity [27,28,29,30] and consume more fruit and vegetable one to two years post-diagnosis or post-treatment [31,32]. However, these longitudinal studies investigated changes in only one of these two behaviors and relied on an examination of patterns of discrete behavior changes (i.e., increase, decrease or no change) at two or three time points. Moreover, most studies have relied on the analysis of between-person associations to support hypotheses that aim to explain how behaviors operate within individuals. Prior research provides limited insight about the direction and magnitude of the temporal relationships (i.e., within-person change) between MVPA and fruit and vegetable intake among cancer survivors. This represents a significant gap because both behaviors operate within-persons [33,34,35] and theories that explain associations between multiple behaviors are inherently within-person. As a result, the levels at which behaviors should be associated (i.e., within-person) and the level at which the covariation between these behaviors has generally been examined (i.e., between-person) is incongruent. This incongruence poses problems because it is not possible to draw conclusion on how these variables are related within-persons from between-person associations [36].

To address this knowledge gap, the current study focuses on the within-person association between daily fruit and vegetable intake and weekly levels of MVPA in women diagnosed with and recently treated for breast cancer. This study has two aims. The first aim was to examine the association between the rates of change in fruit and vegetable intake and in levels of MVPA over a 15-month period following primary treatment completion in women diagnosed with breast cancer. We hypothesized that changes in fruit and vegetable intake would be related to changes in levels of MVPA over time and that these changes would be improvements in behaviors because of common underlying factors leading women to a predisposition to lead a healthy lifestyle (e.g., commitment to staying healthy to prevent cancer recurrence) [24,37]. The second aim was to examine whether daily fruit and vegetable intake and levels of MVPA shortly after treatment completion relate to the rate of change in women’s behaviors over time. It was hypothesized that higher initial levels of MVPA would be associated with greater changes in fruit and vegetable intake and vice versa because higher initial levels of one of the behaviors may facilitate adoption of an additional health-promoting behavior [21,38].

## 2. Materials and Methods

Women who had completed primary treatment for a first breast cancer diagnosis were recruited for a larger study [39]. Inclusion criteria were: ≥18 years of age, ability to read, write, and understand English or French, diagnosed with stage I to III breast cancer, and completed primary cancer treatment (i.e., lumpectomy, single or double mastectomy, chemotherapy, radiation therapy; excluding hormonal treatment) <20 weeks prior to screening. Women were excluded if they had: self-reported health concerns that prevented participation in physical activity, metastatic disease, and/or a previous cancer diagnosis. Participants were recruited through oncologist referrals and print advertisements at local medical clinics and hospitals in Montreal, Canada. Women interested in the study contacted the research team via telephone to obtain additional details about the study and were screened for eligibility.

### 2.1. Procedures

At baseline, eligible participants visited the research lab at McGill University to complete a questionnaire, have their height and weight measured, and receive an accelerometer that they were instructed to wear for the next 7 days. Involvement in this study included completing a food frequency questionnaire to assess fruit and vegetable intake and wearing an accelerometer to measure moderate-to-vigorous intensity physical activity for 7 consecutive days for five data collection periods every 3 months over a 12-month period. Ethics approval was obtained from appropriate University and Hospital Ethics Committees before recruitment, and informed consent obtained from women before data collection. Details about the full study protocol are published elsewhere [39].

### 2.2. Measures

Fruit and Vegetable Intake: a six-item food frequency questionnaire was used to assess fruit and vegetable intake. The items for daily fruit and vegetable intake were preceded by the question: ‘How many times per day, week or month do you…’ (1) drink 100% juice; (2) eat fruit; (3) eat a green salad; (4) eat potatoes; (5) eat carrots; and (6) eat other vegetables. The items used have been validated against three 24-hour recalls and found to be a good proxy of fruit and vegetable servings in a sample of adults 18–64 years of age [40]. In that study, the mean total intake of fruit and vegetables for the two measurement instruments were similar (4.6 servings/day vs. 4.8 servings/day; *p* = 0.92), and the Spearman correlation coefficient for the association between the food frequency questionnaire scores and the 24-h recalls was 0.41 [40]. In this study, fruit and vegetable intake was analyzed as number reported per day (servings/day).

MVPA: participants wore a GT3X accelerometer (Actigraph, Pensacola, FL, USA) for 7 consecutive days to assess weekly levels of MVPA. Participants were instructed to wear the accelerometer on their waist from awakening to bedtime, except during water activities (e.g., bathing, showering, swimming). This device does not have a digital display screen, so no feedback on physical activity was provided to participants. Physical activity counts were captured in 30-s epochs throughout the day and extracted in 60-second intervals then downloaded to a computer file. Based on established cut-points [41], corresponding metabolic equivalent of tasks (MET) values for moderate intensity physical activity ranged from 3.00 to 5.99 METs (1952–5724 counts/min) and MET values for vigorous intensity physical activity were ≥6.00 METs (≥5725 counts/min). Average weekly MVPA duration (min/week) were then calculated. Compliance with wearing the accelerometers was excellent in this study (median = 7 days; range = 4 to 7 days). Scores derived from ActiGraph devices using the Freedson MET model (developed using walking and running activities) are accurate for predicting energy expenditure and time spent in physical activities such as walking (range of 2–5 mph) and running on a flat surface (~5.6 mph; 0% slope) [42,43,44].

Demographic and Health-Related Variables: participants were asked questions regarding their age, breast cancer stage, treatments received, income, ethnicity, marital status, and highest level of education attained. Height and weight were measured by a trained staff at baseline. Body mass index (BMI) was calculated as weight (kg) divided by height in meters squared (m^2^).

### 2.3. Data Analysis

Descriptive statistics (e.g., means, standard deviations, and frequencies) were computed using SPSS version 24 (IBM Corporation, Armonk, NY, USA). A structural equation modeling latent growth modeling (LGM) approach was used to address both research aims using Mplus version 7.31 (Muthén and Muthén, Los Angeles, CA, USA). The robust maximum likelihood (MLR) estimator was used to account for observations missing for MVPA (Time 1 = 1.5%; Time 2 = 9.0%; Time 3 = 9.0%; Time 4: = 10.0%; Time 5 = 9.5%) and fruit and vegetable intake (Time 1 = 2.5%; Time 2 = 11.1%; Time 3 = 14.6%; Time 4 = 19.6%; Time 5 = 22.6%), and to account for potential non-normality and non-independence of observations over time. Several steps were involved. First, two univariate unconditional LGMs were tested to determine the average intercept (i.e., baseline levels) and linear slope values (i.e., rate of linear change) for MVPA and fruit and vegetable intake, as well as the variance around those averages (Models 1a–1b). In these models, loadings were fixed to 1 for the intercept factor, and to 0, 1, 2, 3, and 4 for linear slope factor. Second, both univariate unconditional LGMs were combined into a bivariate unconditional LGM (Model 2) to examine the relationship between the rates of change in levels of MVPA and fruit and vegetable intake (aim 1), and the relationship between the intercepts and slopes of the behaviors (aim 2). Model 2 was statistically adjusted for age, stages of breast cancer, time since diagnosis, level of education, and BMI. In addition to the MLR chi-square (MLRχ2) value, model fit was evaluated by inspecting four goodness-of-fit indices: (1) comparative fit index (CFI; [45]), (2) Tucker–Lewis non-normed index (TLI; [46], (3) root mean square error of approximation (RMSEA; [47]) with its associated 90% confidence interval (CI), and (4) standardized root mean square residual (SRMR; [48]). Based on rule of thumb [48,49], models with a non-significant MLRχ^2^, CFI and TLI values ≥0.95, RMSEA values ≤0.06, and SRMR values ≤0.08 were considered to indicate acceptable fit. Parameter estimates and statistical significance of the means and variances were also examined.

## 3. Results

The analytical sample included 199 women who were enrolled at baseline (Time 1); 154 remained at Time 5 (study retention = 76.6%). Participants’ characteristics and the descriptive statistics for the study variables are presented in Table 1.

The fit statistics for univariate unconditional LGM (Model 1a) for MVPA (min/week) were: MLRχ^2^(14) = 20.92, *p* = 0.10, CFI = 0.97, RMSEA [90% CI] = 0.05 [0.00, 0.09], TLI = 0.98, SRMR = 0.09, suggesting an acceptable fit (with the exception of the SRMR value). Based on the estimated parameters, the average intercept was significantly greater than zero (*M*intercept = 110.48, *p* < 0.001), and the average rate of linear decrease over time was significant (*M*slope = −4.60, *p* < 0.001). There was also significant variance around intercept (5918.74, *p* < 0.001) and the linear slope (133.83, *p* < 0.001), indicating individual differences in both the starting point and rate of change in weekly levels of MVPA.

The fit statistics for the univariate unconditional LGM (Model 1b) for fruit and vegetable intake (servings/day) were: MLRχ^2^(14) = 16.22, *p* = 0.30, CFI = 0.98, RMSEA [90% CI] = 0.03 [0.00, 0.08], TLI = 0.99, SRMR = 0.13, suggesting an acceptable fit (with the exception of the SRMR value). The average intercept was significantly greater than zero (*M*intercept = 6.23, *p* < 0.001), and the average rate of linear decrease over time was significant (*M*slope = −0.14, *p* = 0.002). There was also significant variance around intercept (3.59, *p* < 0.001), but not around the linear slope (0.07, *p* = 0.37), indicating individual differences only in the starting point in fruit and vegetable intake whereas similarity across individuals on the rate of decrease in fruit and vegetable intake.

Next, a multivariate LGM (Model 2), which included intercept and slope factors for levels of MVPA and fruit and vegetable intake, was estimated to examine the relationship between these two behaviors over time (aim 1), as well as the relationship baseline levels of fruit and vegetable intake and MVPA and the rates of change in these two behaviors (aim 2). In this model, the intercepts for levels of fruit and vegetable intake and of MVPA were allowed to covary. The residuals of levels of fruit and vegetable intake and MVPA across time were also allowed to covary as errors across measures were expected to be related. The fit statistics were: MLRχ^2^(71) = 79.06, *p* = 0.24, CFI = 0.99, RMSEA [90% CI] = 0.02 [0.00, 0.05], TLI = 0.99, SRMR = 0.05, suggesting an acceptable fit. The linear slope factor of levels of MVPA was not associated with the linear slope factor of fruit and vegetable intake (unstandardized coefficient = 0.89, standard error [SE] = 0.53, *p* = 0.09). In addition, the intercept of levels of MVPA was not associated with the intercept of fruit and vegetable intake (unstandardized coefficient = 23.35, standard error [SE] = 15.04, *p* = 0.12), the association between the intercept of levels of MVPA and the linear slope factor of fruit and vegetable intake was not significant (unstandardized coefficient = 0.000, standard error [SE] = 0.001, *p* = 0.82), and the association between the intercept of fruit and vegetable intake and the linear slope factor of levels of MVPA was not significant (unstandardized coefficient = −0.29, standard error [SE] = 0.77, *p* = 0.71). It can be inferred from these findings that baseline fruit and vegetable intake and baseline levels of MVPA were not related to the way the other behaviors changed over time. Last, the association between the intercept of levels of MVPA and the linear slope factor of levels of MVPA was significant (unstandardized coefficient = −0.10, *p* < 0.001), meaning that participants with the lowest levels of MVPA at baseline showed greater decreases in MVPA across the study period; this association was not significant for fruit and vegetable intake (unstandardized coefficient = −0.05, *p* = 0.26).

## 4. Discussion

The first aim of this study was to examine whether there was an association between rate of change in daily fruit and vegetable intake and weekly levels of MVPA over a 15-month period following primary treatment completion in women diagnosed with breast cancer. Fruit and vegetable intake and levels of MVPA both decreased across the study period. Contrary to the hypothesis, the rate of change in fruit and vegetable intake was not associated with the rate of change in levels of MVPA. The second aim of this study was to determine whether MVPA and fruit and vegetable intake at study baseline was related to the rate at which women’s other behavior changed over time. Initial levels of MVPA were not significantly associated with rate of change of daily fruit and vegetable intake; likewise, initial fruit and vegetable intake was not associated with rate of change in levels of MVPA. A key implication of these findings is that behavioral interventions promoting fruit and vegetable intake should not be assumed to yield concomitant effects in promoting MVPA or vice versa.

The co-occurrence of low fruit and vegetable intake and physical inactivity noted in previous studies [1,5] suggests that fruit and vegetable intake is positively associated with levels of MVPA among cancer survivors. However, these studies focus on the cross-sectional, between-person association, which does not guarantee a similar within-person association over time [33,34,35]. The results of the present study revealed that previously observed between-person associations may not be mirrored longitudinally, nor at the within-person level. That is, initial levels and change in one of these behaviors within-persons may not predict nor relate to change in the other behavior within-persons. There have been few attempts to examine whether samples of adults (18 to 64 years) without a specific health condition engaged in more physical activity than usual on occasions when they also consumed more fruit and vegetable than usual, or vice versa [33,34,35]. For example, whilst Woolcott et al. [35] found that adults who reported, on average, higher physical activity also reported higher fruits and vegetables intake (*r* = 0.30), change in physical activity was not associated with change in fruits and vegetables over a period of 24 months (*r* = 0.03). The current observation is also consistent with those reported by others, whereby the rate of change in either fruit and vegetable intake and MVPA was not associated with the rate of change in the other behavior over 6-month [34] and 24-month [35] periods. Consideration of both within- and between-person level associations is necessary in future studies as associations at both levels have different implications for the design of interventions [36].

Although the evidence presented here suggests these two behaviors are not changing simultaneously at the same rate in a sample of breast cancer survivors within the first 15 months post-treatment, both behaviors tend to decrease from 3 months post treatment (baseline; Time 1) to 15 months post-treatment (Time 5). This is problematic because worsening diet and physical activity may be associated with a host of health outcomes, including but not limited to weight gain, cardiovascular disease, and cancer recurrence [9,10,11,12,13]. Although the findings with respect to physical activity are consistent with prior research [26,27,30], they are not with respect to fruit and vegetable intake. In fact, empirical evidence provide support for an increase in consumption of fruit and vegetables post-cancer diagnosis and treatment [25,31,32]. A number of potential motivational, emotional, and contextual factors might explain the pattern of health behavior change [21,38], and additional research is needed to understand why these behaviors may change during this early period of survivorship.

Although the behaviors were not related, there was a tendency for those who were less active at baseline to decrease their levels of physical activity more quickly over time, suggesting that breast cancer survivors with low levels of MVPA shortly after completing treatment may be especially susceptible to becoming less physically active. In contrast, baseline fruit and vegetables consumption was not associated with rate of change of fruit and vegetable intake over time. It is unclear if this is due to the relatively high fruit and vegetable intake reported at baseline (*M*intercept = 6.23 serving/day) or the fact that a food frequency questionnaire may have limited capacity to detect changes in dietary behaviors over time. Taken together, it can be inferred from the current study that intervening on physical activity behavior soon after breast cancer treatment should be a priority among breast cancer survivors, especially among those with already low levels of MVPA. In parallel, the study provides weaker support for higher precedence for the promotion of fruit and vegetable intake. Moreover, interventions that target one of these two behaviors (either fruit and vegetable intake or MVPA) may not expect to see an associated change in the other behavior, especially within the first 15 months post-treatment.

There are limitations to the current study that should be considered when interpreting the findings. First, participants were drawn from a convenience sample of volunteer women who were largely White and well educated. These demographic characteristics may limit the generalizability of these findings. Second, the use of a food frequency questionnaire to assess fruit and vegetable intake may have impaired the ability to detect meaningful changes over time in this behavior. Evidence supporting the responsiveness to change (i.e., the ability to detect meaningful changes in fruit and vegetable intake) of food frequency questionnaires remains limited (but see [50] for an exception). However, evidence for acceptable within-person variation in fruit and vegetable intake under conditions of true stability (e.g., test-retest reliability coefficients ranging from 0.50 to 0.70) for food frequency questionnaires has been reported in samples of women with [50] and without [51,52] cancer. With respect to levels of MVPA, the intraclass correlation coefficient was obtained in a laboratory setting (i.e., technical reliability) for activity counts measured with the GT3X ActiGraph for movement that are common to most types of daily activity (from 0.5 to 5524 counts/min) and the three axes was 0.97 [53]. Moreover, BMI and level of education were included in the multivariate LGM, two factors known to be associated with a differential reporting error of food intake [54]. Given the study design, sample size, and available validity evidence, the use of a self-report food frequency questionnaire was deemed more cost-effective than the use of biomarkers of fruit and vegetable intake [55]. We also acknowledge that the GT3X accelerometer is limited in measuring isometric muscle activities or any activities involving movements above the waist (e.g., weightlifting, carrying a load, and pushing). Lastly, the GT3X accelerometer is not waterproof. Hence, study participants were asked to remove the accelerometer when engaging in water-based activities (e.g., swimming, aqua fitness classes, aquabiking). Thus, the accelerometer used in this study did not measure MVPA performed in water.

## 5. Conclusions

In the current study, the rate of change in fruit and vegetable intake was not associated with the rate of change in levels of MVPA (or vice-versa). The results of this study also suggest that baseline levels of either fruit and vegetable intake or levels of MVPA were not associated with rate of change in the other behavior over time. Interventions that target one of these behaviors (either fruit and vegetable intake or MVPA) may not expect to see an associated change in the other behavior, as may have been suggested by previous cancer survivorship research that has mostly focused on the between-person association between fruit and vegetable intake and levels of MVPA. Nonetheless, the findings suggest that intervening on both physical activity behavior and diet soon after treatment completion are meaningful priorities among breast cancer survivors in order to prevent both cancer recurrence and cardiovascular disease. Moreover, the current results highlight the importance of considering the level of analysis (i.e., within- or between-person) when seeking to answer questions about associations between behaviors.

## Figures and Tables

**Table 1:** Sample descriptive characteristics (*N* = 199).

Variables	Mean (Standard Deviation)/Percentage (%)
Age (year)	55.01 (10.96)
BMI ^1^ (kg/m^2^)	26.31 (5.65)
Time since diagnosis (month)	10.62 (3.43)
Time since treatment (month)	3.46 (2.33)
Level of education	
Incomplete high school	11 (5.5%)
High school diploma	48 (24.1%)
College/technical diploma	39 (19.6%)
University degree	101 (50.8%)
Breast cancer stage	
I	83 (41.7%)
II	78 (39.2%)
III	38 (19.1%)
Chemotherapy (yes)	128 (64.3%)
Radiotherapy (yes)	176 (88.4%)
Surgery	
Single mastectomy (yes)	56 (28.1%)
Double mastectomy (yes)	34 (17.1%)
Lumpectomy (yes)	119 (59.8%)
Hormonal therapy (yes)	101 (50.8%)

^1^ BMI: Body Mass Index.

## Data Availability

The data presented in this study are available on request from the corresponding author.
